# Genomic characterization of *bla*_OXA-48_ –carrying IncL plasmids in *Enterobacterales* in Vietnam

**DOI:** 10.17912/micropub.biology.001603

**Published:** 2025-10-14

**Authors:** Linh Dieu Tran, Duy Thai Pham, Nhu Duong Tran, Duc Anh Dang, Hai Anh Tran, H Rogier van Doorn, Keigo Shibayama, Hoang Huy Tran, Masato Suzuki

**Affiliations:** 1 National Institute of Hygiene and Epidemiology, Hanoi, Vietnam; 2 Oxford University Clinical Research Unit (OUCRU), National Hospital for Tropical Diseases, Hanoi, Vietnam; 3 Graduate School of Medicine, Nagoya University, Nagoya, Japan; 4 Antimicrobial Resistance Research Center, National Institute of Infectious Diseases, Japan Institute for Health Security, Tokyo, Japan

## Abstract

Carbapenem-hydrolyzing β-lactamase (carbapenemase) genes have been disseminated among bacteria worldwide; however, only a few instances of bacterial isolates harboring the carbapenemase gene *bla*_OXA-48_ have been reported in Vietnam. Here we report the first genetic characterization of *bla*_OXA-48_ –harboring *Enterobacterales* isolates in Vietnam. Three isolates— *Enterobacter hormaechei* , *Klebsiella pneumoniae* , and *Escherichia coli* —were clinically collected in Hanoi between 2010 and 2015. Whole-genome sequencing revealed that these isolates carried *bla*_OXA-48_ on nearly identical IncL plasmids, closely related to globally disseminated pOXA-48a-like plasmids. Our findings highlight the potential spread of highly transmissible *bla*_OXA-48_ –carrying plasmids in Vietnam, emphasizing the importance of continued surveillance.

**Figure 1. Structural comparison of the indicated bla OXA-48 –carrying IncL plasmids in Enterobacterales isolates ( E. hormaechei NIHE10-0087, K. pneumoniae NIHE12-0848, and E. coli NIHE15-1569 in Vietnam in this study, and K. pneumonia e 11978 in Turkey), and genetic context of the plasmid region surrounding the bla OXA-48 gene on pNIHE10-0087_oxa:**
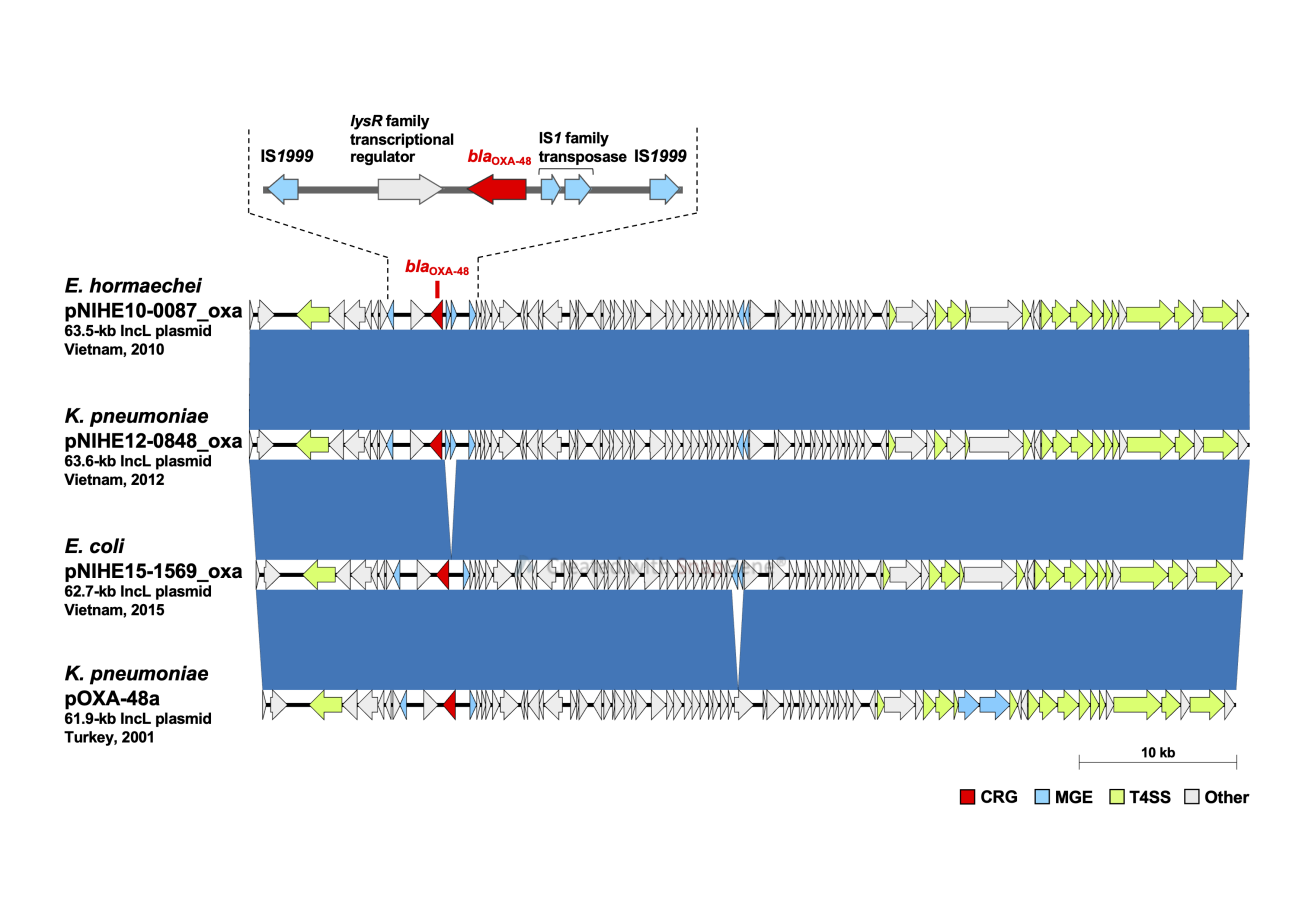
The carbapenem resistance gene (CRG), mobile gene elements (MGE), type IV secretion system-associated conjugation genes (T4SS), and other coding sequences (Other) are highlighted by red, light blue, light green, and gray, respectively. Blue synteny blocks indicate 100 % nucleotide sequence identity.

## Description

Carbapenem-hydrolyzing β-lactamase (carbapenemase) genes confer resistance to a broad spectrum of β-lactams, including third-generation cephalosporins and carbapenems. Carbapenemase genes are often carried on mobile genetic elements (MGEs), such as plasmids, enabling their transfer among Gram-negative bacteria. Carbapenemase-producing *Enterobacterales* (CPE) are a global public health threat due to the limited treatment options for severe infections and high mortality. Among common carbapenemase genes (including *bla*_NDM_ , *bla*_KPC_ , *bla*_IMP_ , and *bla*_OXA-48_ ), *bla*_NDM_ and *bla*_KPC_ are most widely disseminated among *Enterobacterales* in Vietnam (Linh et al., 2021), but the actual status of *bla*_OXA-48_ remains unknown. To date, several variants of OXA-48-like carbapenemase genes, such as *bla*_OXA-48_ , *bla*_OXA-181_ , and *bla*_OXA-232_ , have been identified (Pitout et al., 2019). OXA-48-like carbapenemase genes are thought to be originally encoded on the chromosomes of environmental bacteria, such as *Shewanella* spp., and have been transmitted to clinical relevant bacterial pathogens by plasmids or other MGEs.

Here, we performed plasmid analysis on *bla*_OXA-48_ –harboring *Enterobacterales* isolated in Vietnam. Specifically, we characterized the genetic structures of *bla*_OXA-48_ –carrying IncL plasmids in clinical isolates of *Enterobacter hormaechei* , *Klebsiella pneumoniae* , and *Escherichia coli* , and revealed their similarity with widespread pOXA-48a-like plasmids. These are the first *bla*_OXA-48_ carrying plasmids from Vietnam to be characterized.

We conducted genomic epidemiological studies on CPE isolated in Vietnam (Linh et al., 2021), and identified three *bla*_OXA-48_ –harboring *Enterobacterales* isolates, *Enterobacter cloacae* complex NIHE10-0087, *Klebsiella pneumoniae* NIHE12-0848, and *Escherichia coli* NIHE15-1569. In this study, we analyzed the draft genome sequences of NIHE10-0087, NIHE12-0848, and NIHE15-1569 using the Illumina short-read sequencing platform, and found that they consist of 140 contigs (5.0 Mb, accession no. BPUF01000000), 104 contigs (5.9 Mb, accession no.BPUG01000000), and 148 contigs (5.1 Mb, accession no. BPUH01000000), respectively. ANI analysis revealed that NIHE10-0087, NIHE12-0848, and NIHE15-1569 are 98.8%, 98.9%, and 97.0% identical to *Enterobacter hormaechei* subsp. *steigerwaltii* DSM 16691 ^T^ (accession no. CP017179), *K. pneumoniae* subsp. *pneumoniae* DSM 30104 ^T^ (accession no. AJJI00000000), and *E. coli* DSM 30083 ^T^ (accession no. AGSE00000000), respectively, thereby identifying their species based on the ≥95% ANI threshold. MLST using the schemes for *E. cloacae* , *K. pneumoniae* , and *E. coli* revealed that NIHE10-0087, NIHE12-0848, and NIHE15-1569 belong to sequence types 93 (ST93), 231 (ST231), and 457 (ST457), respectively. These isolates co-harbored clinically important AMR genes, such as another carbapenemase gene *bla*_NDM-1_ in NIHE10-0087 and the extended spectrum β-lactamase (ESBL) gene *bla*_CTX-M-15_ in NIHE12-0848, along with *bla*_OXA-48_ . NIHE12-0848 and NIHE15-1569 were phenotypically susceptible to imipenem and meropenem, which is consistent with a previous finding that OXA-48–producing *Enterobacterales* could test susceptible to carbapenems (Pitout et al., 2019).

To determine the genetic background of *bla*_OXA-48_ we performed long-read sequencing analysis using MinION. The results showed that *bla*_OXA-48_ is located on a 63.5-kb plasmid in NIHE10-0087 (pNIHE10-0087_oxa, accession no. AP025037), on a 63.6-kb plasmid in NIHE12-0848 (pNIHE12-0848_oxa, accession no. AP025038), and on a 62.7-kb plasmid in NIHE15-1569 (pNIHE15-1569_oxa, accession no. AP025039), respectively (Fig. 1). These plasmids carried no known AMR genes other than *bla*_OXA-48_ , commonly had an IncL replicon, and were highly identical: pNIHE12-0848_oxa and pNIHE15-1569 had 99% identity with pNIHE10-0087_oxa, respectively (Fig. 1). BLAST analysis revealed the presence of numerous *bla*_OXA-48_ *–* carrying IncL plasmids, identical to these plasmids in the NCBI database. For example, these plasmids were highly identical to the 61.9-kb *bla*_OXA-48_ *–* carrying IncL plasmid, pOXA-48a (accession no. JN626286) (Poirel et al., 2012), in *K. pneumoniae* 11978 clinically isolated in Turkey in 2001 (99% identity with pNIHE10-0087_oxa) (Fig. 1). According to the antimicrobial susceptibility testing results, *K. pneumoniae* 11978 was also susceptible to carbapenems (Poirel et al., 2012). *bla*_OXA-48_ in all three plasmids in this study was flanked by an IS *1999* element as analyzed in that in pOXA-48a (Hamprecht et al., 2021) (Fig. 1).

pOXA-48a-like IncL plasmids are highly transmissible among *Enterobacterales* and have been detected in *Enterobacterales* isolates around the world (Pitout et al., 2019). These plasmids did not contain additional AMR genes other than *bla*_OXA-48_ (Pitout et al., 2019; Poirel et al., 2012) but have been reported to be associated with increased virulence (Hamprecht et al., 2021). Although this is the first report of pOXA-48a-like IncL plasmids in Vietnam to the best of our knowledge, *bla*_OXA-48_ has also been detected in Vietnam in other studies (Tada et al., 2017). Thus, we speculate that they have been circulating in Vietnam. Continued monitoring of CPE in clinical and environmental settings is required in the future due to the high transmissibility of such plasmids.

**Nucleotide sequence accession nos.**

Draft genome sequences of *E. hormaechei* NIHE10-0087, *K. pneumoniae* NIHE12-0848, and *E. coli* NIHE15-1569, and complete sequences of their *bla*_OXA-48_ *–* carrying IncL plasmids pNIHE10-0087_oxa, pNIHE12-0848_oxa, and pNIHE15-1569_oxa have been deposited at GenBank/EMBL/DDBJ under accession nos. BPUF01000000, BPUG01000000, BPUH01000000, AP025037, AP025038, and AP025039, respectively.

**Competing interests**

None declared.

**Ethical approval**

The samples used in this study were taken from the Isolate Bank of National Institute of Hygiene and Epidemiology (NIHE). This study is a part of the main project “Assessing the impact and burden of antimicrobial resistance in Vietnam, genomic characterization and risk factors related to antimicrobial resistance of common bacteria in Vietnam” which was approved by the Institutional Review Board of NIHE. Individual informed consent was waived because of the retrospective nature of this work and because no personal identifiers were collected (IRB code: IRB-VN01057-38/2016).

## Methods

Carbapenem-resistant *Enterobacterales* isolates from medical institutions in Hanoi, Vietnam were sent to the National Institute of Hygiene and Epidemiology, Vietnam as part of an AMR surveillance programme (see Ethics). PCR targeting common carbapenemase genes, including *bla*_KPC_ , *bla*_NDM_ , *bla*_IMP_ , and *bla*_OXA-48_ , was performed as described previously (Linh et al., 2021). Three *bla*_OXA-48_ –harboring *Enterobacterales* isolates, *Enterobacter hormaechei* NIHE10-0087, *Klebsiella pneumoniae* NIHE12-0848, and *Escherichia coli* NIHE15-1569 were obtained in 2010, 2012, and 2015, respectively. Whole-genome sequencing was performed on these isolates using MiniSeq (Illumina) with MiniSeq High Output Reagent Kit (300 cycles) and MinION (Oxford Nanopore Technologies [ONT]) with the R9.4.1 flow cell. The Illumina sequencing library (paired-end, insert size 500–900 bp) was prepared using the Nextera XT DNA Library Prep Kit, while the ONT sequencing library was prepared using the Rapid Barcoding Kit (SQK-RBK001). De novo assembly of Illumina reads was performed using Shovill v1.1.0 (https://github.com/tseemann/shovill) with default parameters to generate draft genome sequences. ONT reads were basecalled using Albacore v2.3.4. Hybrid de novo assembly with both Illumina and ONT reads was performed using Unicycler v0.4.8.0 (https://github.com/rrwick/Unicycler) with default parameters to generate complete plasmid sequences.

Genome annotation and average nucleotide identity (ANI) analysis were performed on the DFAST server (https://dfast.nig.ac.jp). Multilocus sequence typing (MLST) was performed using MLST v2.0, while antimicrobial resistance (AMR) genes and plasmid replicons were detected by ResFinder v.4.1 and PlasmidFinder v.2.1 with default parameters, respectively, on the CGE server (http://www.genomicepidemiology.org). Type IV secretion system (T4SS)-associated conjugation genes were detected by TXSScan (https://research.pasteur.fr/en/tool/txsscan-models-and-profiles-for-protein-secretion-systems/) with default parameters. Linear comparison of plasmid sequences was performed by BLAST and visualized by Easyfig v2.2.2 (http://mjsull.github.io/Easyfig/).
